# Tip1/CLIP-170 Protein Is Required for Correct Chromosome Poleward Movement in Fission Yeast

**DOI:** 10.1371/journal.pone.0010634

**Published:** 2010-05-13

**Authors:** Sherilyn Goldstone, Céline Reyes, Guillaume Gay, Thibault Courthéoux, Marion Dubarry, Sylvie Tournier, Yannick Gachet

**Affiliations:** LBCMCP, CNRS, Université de Toulouse, Toulouse, France; Texas A&M University, United States of America

## Abstract

The plus-end microtubule binding proteins (+TIPs) play an important role in the regulation of microtubule stability and cell polarity during interphase. In *S. pombe*, the CLIP-170 like protein Tip1, together with the kinesin Tea2, moves along the microtubules towards their plus ends. Tip1 also requires the EB1 homolog Mal3 to localize to the microtubule tips. Given the requirement for Tip1 for microtubule stability, we have investigated its role during spindle morphogenesis and chromosome movement. Loss of Tip1 affects metaphase plate formation and leads to the activation of the spindle assembly checkpoint. In the absence of Tip1 we also observed the appearance of lagging chromosomes, which do not influence the normal rate of spindle elongation. Our results suggest that *S. pombe* Tip1/CLIP170 is directly or indirectly required for correct chromosome poleward movement independently of Mal3/EB1.

## Introduction

Over the last decade the convergent efforts of molecular genetics and live microscopy have revealed a number of proteins that localize to the more dynamic “plus” ends of the microtubules. In living cells, observation of these proteins as GFP fusions has revealed that they are often associated with the control of microtubule dynamics (growing, shrinking or pausing). This well-conserved family of proteins, called the plus-end microtubule tracking proteins (+TIPs), includes CLIP-170, an endosome-microtubule linker [1] known as Tip1 in *S.pombe* [2] or Bik1 in *S.cerevisiae* [3] [4], as well as the EB1 protein (Mal3 in *S.pombe*, Bim1 in *S.cerevisae*) [5] [6]. In fission yeast, growing microtubules emerging from the cell centre are regulated at their plus-end by a +TIP complex including Tip1, Mal3 and the kinesin Tea2 [2], [7], [8].

To ensure faithful transmission of chromosomes during mitosis, sister chromatids must be correctly segregated between the daughter cells. For this to occur each kinetochore must form bivalent attachments with the spindle microtubules and the mitotic apparatus must orient correctly with respect to the division plane of the cell [9], [10]. Failure of either kinetochore attachment or spindle positioning may result in chromosome loss and thus aneuploidy [11], [12]. In order to achieve correct bivalent attachment of the sister chromatids to the spindle, the plus-ends of the microtubules emanating from each pole must interact with, and become embedded in, the kinetochores of each sister pair. Once bivalent attachment is achieved, cohesin and cyclin B are degraded, allowing the segregation of the sister chromatids to the two SPBs. The spindle assembly checkpoint (SAC) monitors the interactions between the kinetochores and the microtubules and delays the onset of anaphase until correct bipolar attachment is established [11]. The molecular actors of this checkpoint pathway include the Mad1, Mad2, Mad3, and Bub1, Bub3, and Mps1/Mph1 proteins [9], [13], [14], [15], [16]. During mitosis, these proteins are recruited to unattached kinetochores, where they inhibit the activation of the anaphase promoting complex, thus blocking the activation of separase, the destruction of cohesion and cyclin B, and thereby the onset of anaphase, until correct attachment is achieved. The exact nature of the defect in the kinetochore-microtubule interactions sensed by the SAC remains unclear but is known to involve problems in kinetochore/microtubule attachment, or lack of tension between attached sister kinetochores [11], [17], [18].

How the plus-ends of the microtubules recognize and interact with the kinetochores remains to be established but the +TIPs are obvious candidates for this role. In *S. pombe*, Mal3 is implicated in a Bub1-dependent checkpoint that prevents monopolar kinetochore attachment and this action is independent of Mad2 [17]. *S. pombe* cells lacking the +TIP proteins are viable, and thus these cells are able to form a spindle and segregate their chromosomes [2]. This is also true for the asymmetrically dividing budding yeast, which unlike *S. pombe* requires functional astral microtubules for cell viability. However, the authors report a clear defect in spindle positioning in these mutants, due to an inability of astral microtubules to capture cortical cues at the cell cortex, which eventually leads to a synthetic lethal phenotype in a Bik1/Bim1 double mutant [3], [6], [19]. In higher eukaryotes, deletion or inhibition of CLIP-170 function by siRNA treatment produces a mitotic delay, suggesting a mitotic function for this protein [20]. Since it is well established that CLIP-170/Tip1 localizes to the kinetochore in mammalian HeLa cells [21], we decided to investigate the role of +TIPs proteins during mitotic progression. In this report, we provide evidence that the +TIP protein Tip1 affects directly or indirectly the poleward movement of the chromosomes at anaphase onset independently of Mal3.

## Results

### Deletion of Tip1 or Tea2 causes a delay in metaphase that is dependent on the spindle assembly checkpoint

**Figure 1:**
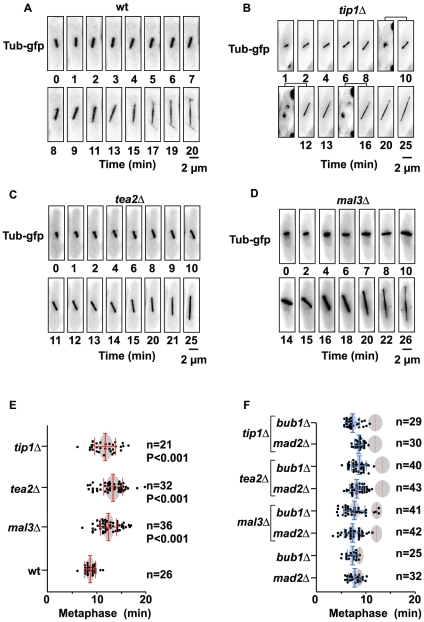
Progression through mitosis in wild-type or in +TIPΔ strains. **A**: Mitotic progression in a representative wild-type cell expressing Atb2-GFP. **B**: Mitotic progression in a representative *tip1*Δ cell. Hoechst staining of the DNA is shown at 10, 12, and 16 minutes. **C**: Mitotic progression in a representative *tea2*Δ cell. **D**: Left panel; Mitotic progression in a representative *mal3*Δ cell. **E**: Statistical analysis of the length of time spent in metaphase in wild-type, *mal3*Δ, *tea2*Δ, and *tip1*Δ cells. The time spent in metaphase was calculated for each cell as the time interval required for the spindle to progress from 2 µm in length to the onset of anaphase B. The means are represented by the red bars. **F**: Statistical analysis of the length of time spent in metaphase in the *mad2*Δ and *bub1*Δ strains, as well as the various double deletions of the +TIP genes and either the *mad2* or *bub1* genes. The red lines represent the mean length of time spent in metaphase in the parental lines (wild-type, *mal3*Δ, *tea2*Δ and *tip1*Δ), while the blue bars represent the mean length of time spent in metaphase in the double delete. The grey circles represent the interval of confidence for each analysis with p<0.05.

While deletion of the *tip1*, *tea2* or *mal3* genes either individually or in combination is not lethal, these mutants all show major defects in interphase microtubule dynamics. It has been previously established that deletion of Mal3 result in mitotic defects [15]. We therefore investigated whether deletion of the Mal3 partners Tea2 or Tip1 also had functional consequences for mitosis. We created strains deleted for *tip1*, *tea2* or *mal3* (as a control), with each strain also carrying a GFP-tagged version of the non-essential α-tubulin gene (Atb2-GFP) in order to follow their progression through mitosis (Figure 1A, B, C, D). We determined the length of time that each of these strains spent in mitosis by live video microscopy, from the time the spindle reached a length of approximately 2 µm up to full spindle elongation. Single focal plane images were collected at 30 sec intervals, and the spindle length was then calculated for each image and plotted as a function of time. The time of entry into anaphase was determined from the plots of spindle length against time and taken to be the time at which rapid spindle elongation began, as has been previously described [22], [23]. We found that wild-type cells spent an average of 8.5+/−0.89 min (n = 26) in metaphase, while *tip1*Δ cells spent an average of 11.7+/−2.1 min (n = 21) in metaphase, with *tea2*Δ cells spending an average of 13.5+/−1.9 min (n = 32) in metaphase (Figure 1E). In the same experimental conditions, we confirmed that *mal3*Δ cells also spent more time in metaphase (12.2+/−1.66 min, n = 36). Statistical analysis (Student's test) revealed that the distribution of the time spent in metaphase in wild-type cells and in cells deleted for *mal3*, *tea2*, and *tip1* was significantly different. It is interesting to note that a metaphase delay was also seen in these strains expressing the kinetochore marker Ndc80-GFP instead of α-tubulin, suggesting that this delay is not due to an increased expression of Atb2-GFP (data not shown).

To determine whether this metaphase delay was dependent on the spindle assembly checkpoint (SAC), we analyzed the time spent in metaphase in strains carrying a deletion of the +TIP gene as well as either of the SAC component genes *mad2* or *bub1*. In each case the deletion of either *mad2* or *bub1*, together with that of *mal3*, *tea2*, or *tip1* abrogated the delay, with the mean time of entry into anaphase returning to that of the wild-type strain (Figure 1F). Thus, the metaphase delay that we observe in the *tip1*Δ and *tea2*Δ strains is primarily dependent on the activation of the SAC, suggesting that these mutants have problems with the establishment of correct bipolar attachment of their kinetochores to the spindle.

### Localization of Tip1 and Tea2 during mitosis

**Figure 2:**
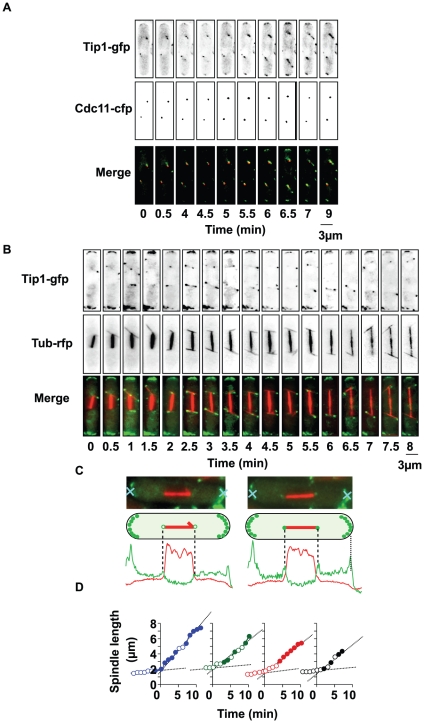
Localization of Tip1 on the mitotic apparatus. **A**: Sequential images of a wild-type cell, expressing Tip1-GFP and Cdc11-CFP (SPB), during mitosis. **B**: Sequential images of mitosis in a wild-type cell co-expressing Tubulin-RFP and Tip1-GFP. **C**: Upper panels; Images of two metaphase wild-type cells co-expressing Tubulin-RFP and Tip1-GFP. Lower panels; Line-scans of the plane through the length of the spindle in early mitosis (left panel) or later in mitosis (right panel). The red lines represent the length of the spindle, while the green traces represent the intensity of the Tip1-GFP signal. **D**: Graphical representation of the timing of appearance of Tip1-GFP at the SPBs during mitosis in four representative cells (Time 0 is anaphase onset). Each trace plots the length of the spindle versus time. Filled circles; Times at which Tip1-GFP is associated with either of the two SPBs.

Given the mitotic defects present in the deletion mutants, we examined whether Tip1, Tea2 or Mal3 were present on the mitotic apparatus. It has been previously reported that Mal3 localizes on the spindle from the time of SPB separation onwards and is also present on astral microtubules during mitosis (Figure S1) [17]. Previous analysis by Brunner and Nurse using immunofluorescence revealed that Tip1 was absent from the mitotic apparatus although it was occasionally seen at the tips of astral microtubules [2]. However, this analysis failed to reveal whether Tip1 also decorated the SPBs and precisely when Tip1 arrived during mitosis. Using live cell analysis of a Tip1-GFP Cdc11-CFP strain (Cdc11 is an SPB protein), we found that Tip1 localized at the tips of the cell (Figure 2A, frames 5 to 9) and as well as on, or close to, the SPBs (Figure 2A). We hypothesized that the presence of Tip1 in the vicinity of the SPBs could be due to its localization on astral microtubules. We constructed a strain carrying both Tip1-GFP and mRFP-tubulin to visualize both Tip1 and the mitotic apparatus. Z-stacks of simultaneously imaged Tip1-GFP and mRFP-tubulin were acquired every 30 seconds. In early mitosis (2 µm spindle), Tip1 did not appear to be present on any element of the mitotic apparatus (Figure 2C, left panel), although we observed Tip1 speckles at the tips of the cell and along the cortex (Figure 2B). However, at later mitotic stages, during spindle elongation, while Tip1 was never seen on the spindle itself, a transient Tip1 signal was seen to accumulate at the SPBs (Figure 2A–D), and decorating the astral microtubules, including their plus ends (Figure 2B). We analysed the timing of appearance of Tip1-GFP at the SPBs during mitosis in four representative cells by plotting the length of the spindle versus time (Figure 2D). The filled circles represent the times at which Tip1-GFP is associated with either of the two SPBs. We found that Tip1 transiently associated with the SPBs, mainly at anaphase onset. The signal transiently present at the SPBs disappeared when cells were treated with high doses of microtubule depolymerizing drugs, confirming that Tip1 associates mainly with astral microtubules rather than the spindle apparatus (data not shown).

We repeated this experimental approach for the localization of Tea2, the kinesin partner of Tip1. Like Tip1, Tea2 associated transiently with the SPBs from mid-mitosis on, and also with the plus ends of the astral microtubules (Figure S2).

**Figure 3:**
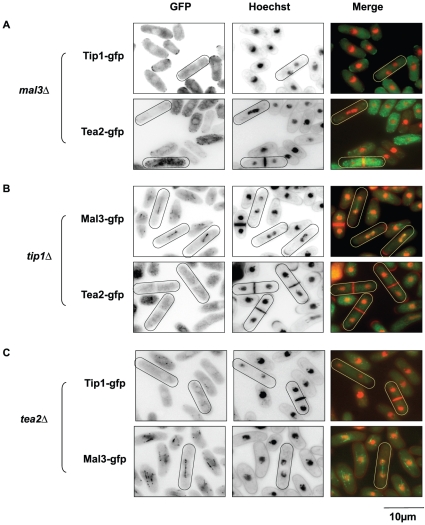
Analysis of the localization co-dependencies of the +TIPs. **A**: Upper panel; Representative field of *mal3*Δ Tip1-GFP cells stained with Hoechst (green, Tip1-GFP; red, Hoechst). Lower panel; Representative field of *mal3*Δ Tea2-GFP cells stained with Hoechst (green, Tea2-GFP; red, Hoechst). **B**: Upper panel; Representative field of *tip1*Δ Mal3-GFP cells stained with Hoechst (green, Mal3-GFP; red, Hoechst). Lower panel; Representative field of *tip1*Δ Tea2-GFP cells stained with Hoechst (green, Tea2-GFP; red, Hoechst). **C**: Upper panel; Representative field of *tea2*Δ Tip1-GFP cells stained with Hoechst (green, Tip1-GFP; red, Hoechst). Lower panel; Representative field of *tea2*Δ Mal3-GFP cells stained with Hoechst (green, Mal3-GFP; red, Hoechst).

Previous studies have shown that the localization of both Tip1 and Tea2 to microtubules is dependent on Mal3 and is largely reduced in *tea2*Δ cells [24]. To understand the mechanism of Tip1 and Tea2 localization on astral microtubules we analyzed their localization in cells deleted for Mal3. Indeed, in *mal3*Δ cells we found virtually no Tip1 signal associated with the SPB and on few astral microtubules (Figure 3A). This was also the case for Tea2 (Figure 3A). In contrast, in both the *tip1*Δ and *tea2*Δ strains, Mal3 localized to the spindle, the residual astral microtubules and the SPBs (Figure 3B, C). However, in *tip1*Δ cells, Tea2 was no longer associated with the microtubules (Figure 3B), nor any component of the mitotic apparatus, despite the presence of Mal3. This was also the case for the localization of Tip1 in *tea2*Δ cells (Figure 3C). These observations suggest that, as is the case during interphase [8], [24], Mal3 is the key component of the mitotic +TIP complex that localizes Tea2 and Tip1 transiently to the SPBs and the astral microtubules.

### Tea2 or Tip1 deleted cells are not sensitive to microtubule destabilizing agents and lose chromosome at a low rate as compared to Mal3

**Figure 4:**
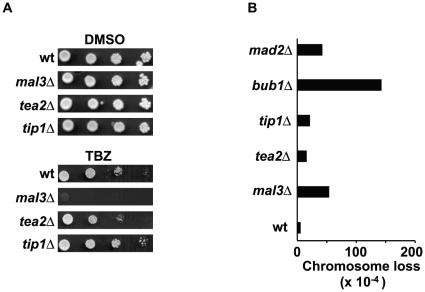
TBZ sensitivity and chromosome loss rate of the +TIPs. **A**: Sensitivity of the *mal3*Δ, *tea2*Δ and *tip1*Δ strains to TBZ. **B**: Rate of chromosome loss in the +TIPΔ strains as compared to wild-type and checkpoint gene delete strains.

Deletion of the SAC component genes *bub1 or mad2* leads to inappropriate mitosis in presence of microtubule destabilizing drugs such as thiabendazole (TBZ) or carbendazime (MBC). This phenomenon has also been observed in cells deleted for Mal3. We tested the sensitivity of *tip1*Δ and *tea2*Δ cells to TBZ and compared it to that of wild type or *mal3*Δ cells. The wild type, *tip1*Δ and *tea2*Δ strains grew equally well at a dose of TBZ of 8 µg/ml, while the *mal3*Δcells were found to be at least 100-fold more sensitive to TBZ than the wild-type cells (Figure 4A).

We were interested to see whether Tip1 or Tea2 deleted cells lose chromosomes at high rate or not. To address this question, we first created *tip1*Δ and *tea2*Δ strains containing a linear minichromosome 16 linked to the *ade* locus [25]. As controls, we also tested the wild-type strain, the *mal3Δ* strain as well as the checkpoint deficient strains *mad2*Δ and *bub1Δ* as controls. A loss frequency of 3.8×10^−4^ was seen in wild-type cells (n = 15 000), whilst in the *mal3*Δ strain the loss frequency was 52×10^−4^ (n = 13 200), a 13.6 fold increase. In the *tip1*Δ and *tea2*Δ strains, the loss frequencies were 19.5×10^−4^ (n = 16 000) and 14×10^−4^ (n = 13 400) respectively, representing increases of 5.1-fold and 3.8-fold over the rate of chromosome loss seen in the wild-type cells (Figure 4B).

As previously reported, deletion of Mal3 adversely affects genome stability and *mal3* delete cells are hypersensitive to microtubule destabilizing agents. In contrast, mitotic progression in *tip1* or *tea2* delete cells results in SAC activation, with a relatively low chromosome loss rate and TBZ sensitivity. This paradox suggests that the microtubule attachment defects present in *tip1* or *tea2* delete cells are somehow different from those in the *mal3* deleted cells.

### Deletions of Mal3, Tea2 or Tip1 give rise to different mitotic phenotypes

**Figure 5:**
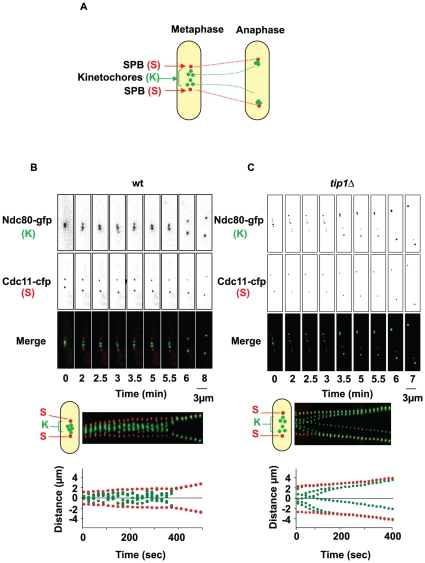
Kinetochore dynamics during mitosis in the wild-type and *tip1*Δ strains. **A**: Each strain carried epitope tagged versions of the core kinetochore component Ndc80 (green, K) and the SPB protein Cdc11 (Cdc11-CFP in red, S). **B**: Upper panel; Sequential images of a wild-type cell expressing Ndc80-GFP and Cdc11-CFP during mitosis. Middle panel; Kymograph representation of the same cell. Lower panel; Graphical analysis of the movements of the SPBs (red) and the kinetochores (green) in this cell, determined by automated analysis. **C**: Upper panel; Sequential images of a *tip1*Δcell expressing Ndc80-GFP and Cdc11-CFP during mitosis. Middle panel; Kymograph representation of the same cell. Lower panel; Graphical analysis of the movements of the SPBs (red) and the kinetochores (green).

**Figure 6:**
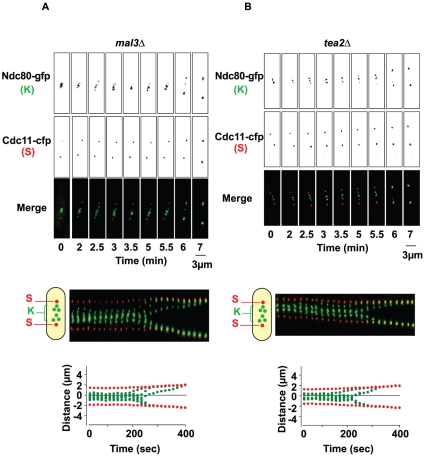
Kinetochore dynamics during mitosis in the *mal3Δ* and *tea2*Δ strains. **A**: Upper panel; Sequential images of a *mal3*Δ cell expressing Ndc80-GFP and Cdc11-CFP during mitosis. Kymograph representation of the same cell. Graphical analysis of the movements of the SPBs (red) and the kinetochores (green). **B**: Upper panel; Sequential images of a *tea2*Δ cell expressing Ndc80-GFP and Cdc11-CFP during mitosis. Middle panel; Kymograph representation of the same cell. Lower panel; Graphical analysis of the movements of the SPBs (red) and the kinetochores (green).

Because loss of Mal3, Tea2 or Tip1 resulted in the activation of the SAC, we investigated the dynamics of sister chromatid movements in the +TIP deletion strains. To this end, we created *mal3*Δ, *tip1*Δ and *tea2*Δ strains carrying a GFP-tagged version of the *ndc80* gene (a kinetochore component; Figure 5A). These strains allowed us to visualize the movements of the kinetochores and SPBs during progression through mitosis. We collected images of mitotic cells as Z-stacks of 3 planes at 0.45 µm intervals every 15 seconds from metaphase until the onset of anaphase for at least 50 mitotic cells. Figures 5 and 6 show representative movies of the phenotypes observed.

In wild-type cells, during metaphase the kinetochores oscillated between the two SPBs, eventually forming a metaphase plate (Figure 5B, frame 5), after which they segregated rapidly to each of the poles as previously described [23], [26]. Interestingly, in *tip1*Δ cells, the kinetochores did not align on a metaphase plate before anaphase onset (Figure 5C; see frames 1 to 4). Additionally, while wild-type cells segregated their chromosomes in a coordinated manner, cells deleted for Tip1 frequently showed lagging chromosomes at anaphase onset. Kymographic representation and automated analysis (see Material and Methods) showed that these lagging chromosomes were visible throughout anaphase B and regained their SPBs with an obviously reduced rate of poleward movement (P-movement) (Figure 5C, lower panels). The same analysis was performed in cells deleted for *mal3* and *tea2* (Figure 6A and 6B). Although we noticed the presence of uncoordinated chromosome segregation in these cells (Figure 6A and B) and the absence of a metaphase plate prior to anaphase onset, we found that the lagging chromosomes in the absence of Mal3 and Tea2 regained their SPBs relatively rapidly as compared to those seen in *tip1*Δ cells (Figure 6A). Interestingly, although chromosome congression is sometimes observed during metaphase in *tea2Δ* cells (Figure 6B, frame 2), we find that it is not always associated with anaphase initiation.

**Figure 7:**
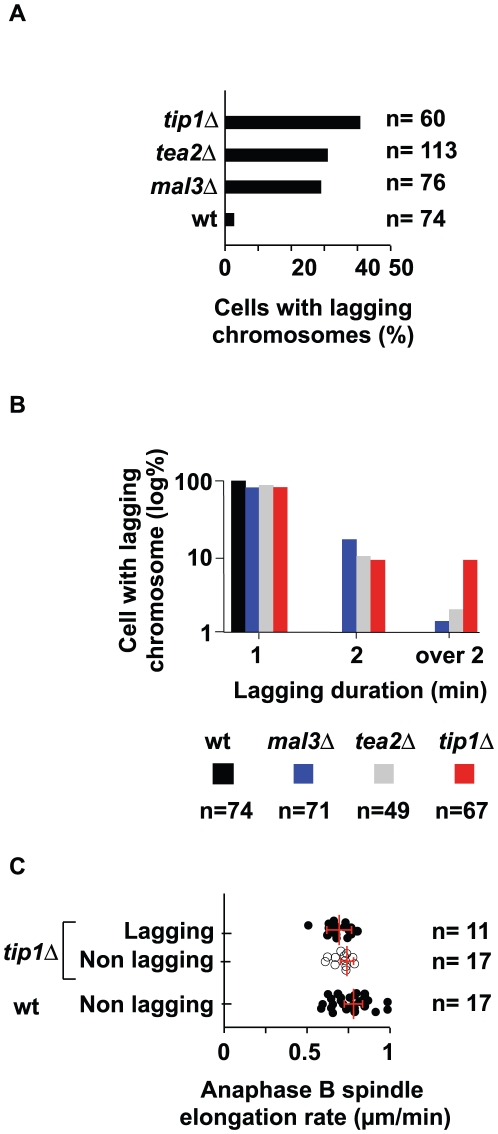
Analysis of chromosome dynamics during mitosis in the +TIPs. **A**: Percentage of cells entering anaphase A with one or more lagging chromosomes. **B**: Repartition of lagging chromosomes as a function of their lag times in a logarithmic scale. **C**: Rate of spindle elongation during anaphase B in the wild type and tip1Δ cells. The empty circles represent mitosis without lagging chromosomes present, while the filled circles represent mitosis with lagging chromosomes. The mean spindle elongation rate (red bars) was calculated for each strain and were not found to be significantly different (student test).

We next quantified the percentage of lagging chromosomes (defined as a delay of at least 30 s after the first kinetochore permanently reaches the pole). In wild type this phenomenon is observed in only 2.15% (n = 74) of cases but reaches 40% (n = 60), 30% (n = 113) and 29% (n = 76) in *tipΔ*, *tea2Δ* and *mal3Δ* cells, respectively (Figure 7A). However, as seen in Figure 6, we found that the length of time that the chromosomes lagged was greatly increased in *tip1Δ* cells as nearly 10% of mitotic cells (n = 67) showed a lagging chromosome duration time of over 2 minutes during anaphase B (Figure 7B). In these cells, the lagging chromosomes were clearly affected in their poleward movement as we found that they regained the pole at a reduced speed of 0.4 µm/min +/−0.2 (n = 8) as opposed to 1.2 µm+/−0.4 (n = 22). To address whether the specific lagging kinetochore phenotype observed in cells deleted for Tip1 was a consequence of the lack of chromosome congression, we quantified the defects in chromosome congression in these different strains.

In wild-type cells only 8% (n = 74) of the cells fail to show kinetochore congression just prior to initiation of anaphase A. In contrast, over 50% of cells initiated anaphase A in the absence of chromosome congression in the +TIP deleted strains (64% n = 71; 50% n = 49; and 55% n = 67 for *mal3*Δ, *tea2*Δ and *tip1*Δ, respectively) (Figure S3). It is possible that when Tip1 is deleted, Mal3, its main partner, associates with a different protein complex, conferring a new mitotic phenotype (ie. a defect in poleward movement). To address whether the specific lagging kinetochore phenotype observed in cells deleted for Tip1 was dependent on the presence of Mal3, (e.g. in the absence of Tip1, Mal3 could associate with and titrate a protein required for poleward movement), we created a double mutant *tip1Δ mal3Δ* and quantified the number of lagging chromosomes present in this strain. We found no significant differences in the rate of poleward movement of the kinetochores in the single *tip1Δ* mutant as compared to the double *tip1Δ mal3Δ* mutant (data not shown). Therefore, we conclude that Tip1 directly or indirectly participates in normal chromosome dynamics at anaphase onset, independently of Mal3.

In *S. pombe*, spindle elongation is determined by the speed of tubulin incorporation at the spindle midzone [27] rather than by the pushing forces of astral microtubules in anaphase B [28]. Furthermore, a recent report has shown that Bim1 (*S. cerevisiae* homologue of Mal3/EB1) provides structural support for spindle elongation [29]. Finally, it has been suggested that lagging chromosomes may affect spindle elongation in *S. pombe* [30]. Indeed, we recently demonstrated that merotelic attachment can mechanically prevent full spindle elongation [12]. However, we did not observed stretched merotelic kinetochores in cell deleted for Tip1 (data not shown), suggesting that the lagging chromosomes seen in this mutant are single sister chromatids, monotelically attached rather than merotelically attached. Additionally, we found no statistically significant differences in the rate of spindle elongation in +TIP mutants whether lagging chromosomes were present (Figure 7D, white circles) or not (Figure 7D, black circles). Therefore our results provide evidence that spindle elongation is specifically reduced in the presence of merotelic kinetochores [12] but not with single sister chromatids monotelically attached. Statistical analysis (Student's test) revealed that the rate of spindle elongation in *tip1Δ* mutants was indistinguishable from that of wild-type cells, suggesting that Tip1 does not play a role in the control of the dynamics of the interdigitated microtubules at the spindle midzone, and that it is not required for normal spindle elongation. Taken together, our observations suggest that Tip1 is required for correct chromosome poleward movement in fission yeast.

### The Mad2 and Bub1 proteins are recruited to the kinetochores in *tip1*Δ cells

**Figure 8:**
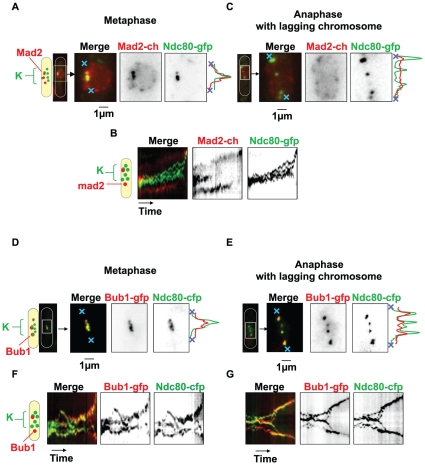
Recruitment of Mad2 and Bub1 to lagging chromosomes in the *tip1*Δ strain. Left-hand panels; Metaphase cells. Right-hand panels; Anaphase cells with lagging chromosomes. **A**: The upper block shows images of an early metaphase *tip1*Δ cell, expressing Mad2-Cherry (red) and Ndc80-GFP (green). The lower block shows a cell in late metaphase. The line-scans of the merged images is shown on the right. The position of the line taken for the scans is marked by the blue crosses. **B**: Kymograph showing the localization of Mad2 during progression through metaphase in a *tip1*Δ Mad2-Cherry Ndc80-GFP cell. **C**: Images of an anaphase *tip1*Δ Mad2-Cherry Ndc80-GFP cell with lagging chromosomes. **D**: Images of a metaphase *tip1*Δ cell, expressing Bub1-GFP (green) and Ndc80-CFP (red). **E**: Images of an anaphase *tip1*Δ Bub1-GFP Ndc80-CFP cell showing lagging chromosomes. **F**: Kymograph showing the localization of Bub1 during progression through metaphase in a *tip1*Δ Bub1-GFP Ndc80-CFP cell. **G**: Kymograph showing the localization of Bub1 during progression through anaphase in a *tip1*Δ Bub1-GFP Ndc80-CFP cell.

Given the SAC-dependent metaphase delay observed in *tipΔ* cells and the presence of lagging chromosomes, we decided to investigate the recruitment of Mad2 and Bub1 to the kinetochores. To do this, we created two new *tip1*Δ strains: *tip1*Δ Ndc80-GFP Mad2-mCherry, and *tip1*Δ Ndc80-CFP Bub1-GFP, to allow us to follow the dynamics of the kinetochores and the checkpoint proteins during progression through mitosis. As expected, both Mad2 and Bub1 were recruited to the kinetochores during prometaphase/early metaphase, with Mad2 leaving the kinetochores in early metaphase and moving to one SPB well before the kinetochores attained their respective poles during anaphase A (n = 50) (Figure 8A), after which the Mad2 signal rapidly disappeared. A kymographic representation of this process is shown in Figure 8B. Interestingly, when lagging chromosomes persisted into anaphase, Mad2 was never seen on the lagging kinetochore but persistently observed at the SPB (n = 70) (Figure 8C). This recruitment pattern mirrored that seen in wild-type cells, although in the *tip1*Δ cells the Mad2 signal tended to persist slightly longer on the kinetochores during early metaphase, reflecting the SAC-dependent delay in this strain. In contrast, in the *tip1*Δ cells, Bub1 was present on the kinetochores throughout metaphase, albeit at a lower level than during prometaphase (n = 50) (Figure 8E and G). At the transition to anaphase A, this residual Bub1 signal moved to the SPBs together with the kinetochores, after which it decayed. Significantly, when lagging chromosomes were present during anaphase, a clear Bub1 signal was associated with certain, but not all, of the lagging kinetochores, persisting at least until the lagging kinetochore reached its pole (n = 60) (Figure 8F and H). These experiments show that the lagging kinetochores showing a defective P-movement are not detected by Mad2 although they can recruit the spindle assembly checkpoint protein Bub1. This result suggests that the lagging chromosomes present in the *tip1*Δ cells are attached (not detected by Mad2) but can still be detected by Bub1, and thus may not be under correct tension.

## Discussion

Our results show that Tip1 has a function in the poleward movement of the chromosomes during mitosis. This function is independent of the presence of its partner Mal3, and does not involve an association between Mal3 and any other protein complex since the *tip1Δ mal3Δ* double mutant still exhibits this poleward movement defect. Deletions of the *mal3*, *tea2* and *tip1* genes, individually or in combination, compromises mitotic progression although as previously observed, the defects are more severe in the case of Mal3 [17]. We show that deletion of the *tip1* or *tea2* genes causes a Mad2 and Bub1-dependent metaphase delay in each case. Interestingly, in higher eukaryotes inhibition of CLIP-170 imposes a Mad2-dependent mitotic delay, in agreement with our observations [31].

In eukaryotic cells, including yeast, the chromosomes align at the spindle midzone during metaphase to form the “metaphase plate” [26], [32], [33], [34]. This process is thought to be mainly dependent on microtubule dynamics although several reports suggest that actin participates in this mechanism in starfish oocytes, *Xenopus* and in *S. pombe* [35], [36], [37]. In fission yeast, formation of the metaphase plate occurs on average 1 minute before the onset of anaphase A. Our analysis of kinetochore dynamics in cells deleted for the +TIPs reveals that anaphase A is initiated while the kinetochores are still randomly positioned on the spindle. Our observations are in agreement with those from studies in higher eukaryotes where RNA interference was used to block the expression of CLIP-170 (HeLa cells) or EB1 (*Drosophila* S2 cells), which in each case prevents normal metaphase plate formation [31], [38]. Given that anaphase onset is initiated randomly, we hypothesize that the distal chromosomes will arrive at the poles later than the proximal chromosomes in the absence of correct congression to the metaphase plate. If this is true, uncoordinated chromosome segregation should be observed, resulting in the presence of lagging chromosomes that regain their SPBs at a normal speed. Indeed, our analysis of kinetochore dynamics in *mal3*Δ and *tea2*Δ cells confirms this hypothesis, i.e. the absence of normal chromosome congression followed by uncoordinated segregation, though with a normal rate of P-movement. However, our study uncovers a specific role for the Tip1 protein in the control of chromosome P-movement that is independent of Mal3 since the lagging chromosomes observed in *tip1Δ* cells are still present in the double mutant *tip1Δ mal3Δ*.

The presence of lagging chromosomes is usually associated with an increased rate of chromosome loss, as has been previously reported for *mal3*Δ cells [15], [17]. Furthermore, *mal3*Δ cells are highly sensitive to low doses of TBZ [15]. The deletion of the budding yeast homolog of Mal3, Bim1, also leads to hypersensitivity to microtubule destabilizing drugs associated with an aberrant spindle morphology [6], [39]. However, we find that deletion of either Tea2 or Tip1 only induces a moderate increase in the rate of chromosome loss and that these cells are not particularly sensitive to TBZ. Therefore it is unlikely that lack of chromosome congression before anaphase onset or a decrease in the rate of chromosome P-movement at anaphase onset is detrimental for the maintenance of genomic stability. We believe that the critical event that is necessary for the prevention of chromosome loss is likely to be correct chromosome bi-orientation. In agreement with this hypothesis, we find that *mal3*Δ cells display an increased rate of chromosome 1 mis-segregation as has been previously described [17] while *tea2*Δ and *tip1*Δ cells do not (data not shown). An alternative explanation for the relatively low rate of chromosome loss in the absence of Tip1 is that late mitotic events such as telophase or cytokinesis may be delayed in these cells, thereby leaving enough time for the eventual retrieval of any lagging kinetochores to the SPBs. Further work will undoubtedly clarify this point.

Given that in higher eukaryotic cells, including *Drosophila* S2 and Hela cells, as well as in *S. cerevisiae*, the Tip1 homologues CLIP-170 and Bik1 are clearly present at the kinetochores [21], [40], [41], [42], a direct role for Tip1 at the kinetochore/microtubule interface would provide the most logical explanation of the mitotic phenotypes observed. Indeed, one possible explanation of the *tip1*Δ and *tea2*Δ mitotic phenotypes could be that these proteins interact directly with the plus ends of the spindle microtubules and regulate catastrophe, as a high frequency of microtubule catastrophe may result in kinetochore/microtubule attachment defects. However, while we were able to confirm the localization of Mal3 on the spindle, we have consistently been unable to detect Tip1 and Tea2 at the kinetochores or on the spindle. This does not exclude the possibility that Tip1 is indeed present at the kinetochores at a level below the detection threshold of our system.

Since Tip1 and Tea2 appear to be absent from the spindle microtubules, this raises the intriguing possibility that Tip1 and Tea2 could influence microtubule dynamics by virtue of their transient interaction with the SPBs or alternatively, their cytoplasmic localization. One possibility is that Tip1 may associate with other proteins within the cytoplasm independently of Mal3 and that loss of this cytoplasmic complex somehow perturbs mitotic progression. A specific role for Tip1 at the SPB is challenged by the fact that Mal3 seems to be required for its localization to spindle. However, Busch et al reported that residual localization of Tip1 on the SPBs is seen in the absence of Mal3 suggesting that Tip1 may have a function at the SPB independently of its main partner, Mal3 [24]. In agreement with our observations, previous studies in budding yeast illustrate the redundant functions of Tip1/Bik1/CLIP-170 and Tea2/Kip2 in spindle morphogenesis [42], [43].

Genetic analysis first suggested that Bik1p was likely to play a role in the dynein pathway [44]. The molecular basis for these genetic observations was elucidated in an elegant series of cell biological studies that showed that Bik1p helps to recruit dynein to the plus ends of the microtubules [45]. We therefore hypothesized that Tip1 could serve as a platform on the SPB for the loading of motor proteins such as Klp2, Klp5 or dynein onto the spindle. Indeed, some of these motor proteins are involved in normal chromosome-to-pole movement in *S. pombe* [23], [46], [47], [48], [49]. However we found that the levels of Klp2 and Klp5 seen on the spindle in cells deleted for Tip1 were comparable to those in wild-type cells (data not shown). Because dynein plays a role in chromosome segregation and bi-orientation in yeast [23], [46], it is possible that some of the phenotypes observed in Tip1 delete cells result from the absence of an interaction with dynein. Further work will be necessary to address this point.

In conclusion, Tip1 appears to play a role during anaphase A that is independent of those of its partners Mal3 and Tea2. The role played by Tip1 on kinetochore P-movement may be either direct or indirect but it represents a new function for a member of the CLIP-170 protein family.

## Materials and Methods

### Yeast strains, growth and maintenance

The strains used in this study are listed in Table 1. Media, growth, maintenance of strains and genetic methods were as described in Moreno et al, (1991). All strains were grown in YES at 25°C until mid-log phase, when aliquots were taken for preparation for processing for live cell imaging, thiabendazole (TBZ) sensitivity assays, or the determination of the rate of chromosome loss.

**Table 1:** Strains used in this study.

Strain	Genotype	Reference
wild-type	lys1::nmt1-atb2GFP leu1-32 ura4-D18	ST7
mal3-GFP	mal3-pk-GFP:ura4+ leu1-32 ura4-D18 ade6-M210 his3-D1	ST123
tip1-GFP	tip1-GFP: kan^R^ leu1-32 ura4-D18 ade6-M216	ST251
tip1-GFP tubulin-RFP	tip1-GFP:kan^R^ leu1-32 ura4-D18 ade6-M216 pREP2-mRFP-atb2	ST567
tea2-GFP	tea2-GFP: kan^R^	ST232
tea2-GFP tubulin-RFP	tea2-GFP: kan^R^ pREP2-mRFP-atb2	ST622
mal3-GFP ndc80-CFP	mal3-pk-GFP:ura4+ ndc80-CFP	ST161
*mal3*Δ tip1-YFP	mal3::ade tip1-YFP:kan^R^	ST330
*mal3*Δ tea2-GFP	mal3::his3+ tea2-GFP:kan^R^ ura4-D18	ST254
*tip1*Δ mal3-GFP	tip1::kan^R^ mal3-pk-GFP:ura4+	ST133
*tip1*Δ tea2-GFP	tip1::kan^R^ tea2-GFP:kan^R^ leu1-32 ade6-M216	ST252
*tea2*Δ mal3-GFP	tea2::his3+ mal3-pk-GFP:ura4	ST162
*tea2*Δ tip1-GFP	tea2::his3+ tip1-YFP:kan^R^ ade6-M210	ST257
*mal3*Δ atb2-GFP	mal3::ura4+ lys1::nmt1-atb2GFP leu1-32 ura4-D18 ade6	ST25
*tip1*Δ atb2-GFP	tip1::kan^R^ lys1::nmt1-atb2GFP ura4-D18 leu1-32	ST109
*tea2*Δ atb2-GFP	tea2::his3+ lys1::nmt1-atb2GFP	ST172
wild-type Ch16	Ch16 ade6-216 leu1-32 ura4-D18 ade6-M210 his1-102	J.P Javerzat
*mal3*Δ Ch16	mal3::ura4+ Ch16 ade6-216 leu1-32 ura4-D18	ST188
*tip1*Δ Ch16	tip1::kan^R^ Ch16 ade6-216 leu1-32 ura4-D18	ST576
*tea2*Δ Ch16	tea2::his3+ Ch16 ade6-216 leu1-32 ura4-D18	ST537
*mad2*Δ Ch16	mad2::ura4+ Ch16 ade6-216 bub1::ura4+ leu1-32 ura4-D18 ade6-M210 his1-102	ST75
ndc80-GFP cdc11-CFP	ndc80-GFP:kan^R^ cdc11-CFP:kan^R^ ura4-D18 leu1-32	ST102
*mal3*Δ ndc80-GFP cdc11-CFP	mal3::ura4+ ndc80-GFP:kan^R^ cdc11-CFP:kan^R^ leu1-32 ura4-D18	ST731
*tip1*Δ ndc80-GFP cdc11-CFP	tip1::kan^R^ ndc80-GFP:kan^R^ cdc11-CFP:kan^R^ ura4-D18	ST655
*tea2*Δ ndc80-GFP cdc11-CFP	tea2::his3+ ndc80-GFP:kan^R^ cdc11-CFP:kan^R^	ST 528
*mad2*Δatb2-GFP	mad2::ura4+	ST653
*bub1*Δ atb2-GFP	bub1::ura4+ lys1::nmt atb2-GFP	ST412
*mal3*Δ *mad2*Δ atb2-GFP	mal3::his3+ mad2::ura4+ lys1::nmt-atb2-GFP	ST546
*mal3*Δ *bub1*Δ atb2-GFP	mal3::ura4+ bub1::leu lys1::nmt-atb2-GFP	ST587

### Cell imaging

Time-lapse video microscopy was performed in imaging chambers (CoverWell PCI-2.5, Grace Bio-Labs) filled with 1 ml of 2% agarose (Sigma) in minimal medium and sealed with a 22×22 mm glass coverslip. An aliquot of cell suspension was applied to the imaging chamber and the cells were allowed to equilibrate for 1 hr at 25°C before beginning the experiments, which were carried out at this temperature. Where visualization of the chromosomes was necessary, either 0.1 mg/ml DAPI or 0.1 mg/ml Hoechst (Molecular Probes) was added to the cell suspensions for 20 min. Time-lapse images were taken at 15 to 30 sec intervals, with exposure times of 0.1–0.3 sec (for GFP) or 0.05 sec for (DAPI). In all cases, a single focal plane image was recorded at each time point. Time zero in the movies was taken to be either the point at which the spindle attained 2 µm or became stable in length. Images were collected with a Princeton CoolSnap HQ^2^ CCD camera (Roper Scientific) fitted to a Leica DM6000 microscope equipped with a 100×1.4 NA objective, Semrock filters (GFP band pass width 562–730 nm, CFP band pass width 467–600 nm, DAPI band pass width; 90% transmission and Texas Red 595–664 nm) and a HIGHlite illumination source (Roper Scientific) reduced to 30% intensity to minimise photobleaching and phototoxicity. All images were recorded using Metamorph software, then either downloaded into Adobe Photoshop for assembly into montages, or processed by automated analysis as discussed below. The length of the mitotic spindle was determined using Metamorph software. For the analysis of spindle and kinetochore dynamics, images were acquired as Z-stacks of 3 planes with a step size of 0.45 µm, at 15 sec intervals, starting from a spindle size of approximately 2 µm and continuing to a length of approximately 10 µm. Maximum projections of the images from each time point were downloaded into Adobe Photoshop for assembly into montages, or into NIH ImageJ for preparation of automated data analysis as described below.

### Automated data analysis of kinetochore dynamics

The position of the SPBs and kinetochores were determined by the visualization of the Cdc11-CFP and Ndc80-GFP signals and captured using Metamorph. Maximum intensity projections were prepared for each time point, with the images from each channel being combined into a single RGB image. These images were cropped around the cell of interest, and where necessary optional contrast enhancement was performed in ImageJ. The cropped images were exported to IGOR Pro6 (www.wavemetrics.com) as 8-bit RGB-stacked TIFF files, each frame corresponding to one image of the time-lapse series. The first step of the automated analysis consisted of the detection of the two SPBs by the localization of the two local maxima of the red signal for each frame of the stacked TIFF file. The positions of these maxima were assumed to correspond to the positions of each SPB. The pointing precision was thus limited to one pixel in each direction. To avoid time-consuming procedures, Gaussian fit and centre-of-mass evaluation were not implemented. Manual correction of the detected trajectories was performed if necessary and the spindle length was then calculated from the SPB positions. The second step of the analysis involved the detection of the positions of the kinetochores, by searching for the green signal intensity maxima along the spindle, defined as a straight line between the SPBs. No assumptions were made on the number of such maxima, to account for the possible super-positioning of the kinetochores. The kinetochores and SPB positions were then displayed graphically with respect to the spindle centre. Certain parameters (smoothing coefficient, signal-to-noise ratio and signal threshold) were varied as necessary to obtain the best result in comparison to the original movie. The data generated were used to calculate the average speed at which the kinetochores reached the SPBs (the rate of P-movement).

### Thiabendazole sensitivity assays

All strains to be tested were grown in YES medium (25°C, with agitation at 200 rpm) to mid-log phase, then harvested and re-suspended in YES to a final concentration of 1×10^6^ cells/ml. Serial 1/5 dilutions were prepared from this suspension, giving a range from 1×10^6^ to 8×10^3^ cells/ml. Aliquots of 4 µl of each dilution were then spotted on YES agar plates containing either 0, 2, 4, 6, 8 or 10 µg/ml TBZ (prepared from a 10 mg/ml stock dissolved in DMSO) and allowed to absorb before incubation of the plates at 25°C for a minimum of 3 days, or until the resulting colonies were well grown.

### Minichromosome loss assays

The wild-type, *mal3*Δ, *tip1*Δ, and *tea2*Δ strains, all carrying minichromosome 16, were plated for single colonies on YES containing 12.0 mg/l adenine. Single white colonies were selected for each strain for plating assays. Each colony was re-suspended in 5 ml of YES containing 12.5 mg/l adenine, and the cells were counted. For each strain, a minimum of 5×10^3^ colonies were plated at a density of 500 colonies per 10 cm diameter plate. After 3–5 days growth at 25°C, the plates were transferred to a cold-room at 4°C for a further 2 days. The total number of colonies per strain was counted, and then the colonies were further scored as white, red, half-red, quarter-red or less than one quarter-red. The frequency of minichromosome loss was calculated as follows: A/A+B, where A represented the number of colonies that contained a red sector of at least half the size of the colony, and where B represented the number of colonies that where either white, or contained a red sector less than half the size of the colony.

## Supporting Information

Figure S1 Localization of Mal3 on the mitotic apparatus. Upper panel; image series showing the localization of Mal3 on the spindle as cells progress through mitosis. Lower panel; graphic representation of progression through mitosis in this cell, as shown by plotting the length of the mitotic spindle versus time. The dotted red lines represent the slope of the curves. The intersection of the two dotted lines indicates the transition to anaphase B (A) in this cell, since it is at this point that rapid spindle elongation commences.
(0.48 MB EPS)

Figure S2 Localization of Tea2 on the mitotic apparatus. A: Sequential images of a wild-type cell, expressing Tea2-GFP and Cdc11-CFP (SPB), during mitosis. B: Sequential images of mitosis in a wild-type cell co-expressing Tubulin-RFP and Tea2-GFP.
(4.93 MB EPS)

Figure S3 Analysis of the defects in chromosome congression in the +TIPΔ strains.
(0.14 MB EPS)

## References

[1] Pierre P, Scheel J, Rickard JE, Kreis TE (1992). CLIP-170 links endocytic vesicles to microtubules.. Cell.

[2] Brunner D, Nurse P (2000). CLIP170-like tip1p spatially organizes microtubular dynamics in fission yeast.. Cell.

[3] Miller RK, D'Silva S, Moore JK, Goodson HV (2006). The CLIP-170 orthologue Bik1p and positioning the mitotic spindle in yeast.. Curr Top Dev Biol.

[4] Perez F, Diamantopoulos GS, Stalder R, Kreis TE (1999). CLIP-170 highlights growing microtubule ends in vivo.. Cell.

[5] Tirnauer JS, Salmon ED, Mitchison TJ (2004). Microtubule plus-end dynamics in Xenopus egg extract spindles.. Mol Biol Cell.

[6] Schwartz K, Richards K, Botstein D (1997). BIM1 encodes a microtubule-binding protein in yeast.. Mol Biol Cell.

[7] Maekawa H, Schiebel E (2004). CLIP-170 family members: a motor-driven ride to microtubule plus ends.. Dev Cell.

[8] Browning H, Hackney DD, Nurse P (2003). Targeted movement of cell end factors in fission yeast.. Nat Cell Biol.

[9] Przewloka MR, Glover DM (2009). The kinetochore and the centromere: a working long distance relationship.. Annu Rev Genet.

[10] Gachet Y, Reyes C, Goldstone S, Tournier S (2006). The fission yeast spindle orientation checkpoint: a model that generates tension?. Yeast.

[11] Musacchio A, Salmon ED (2007). The spindle-assembly checkpoint in space and time.. Nat Rev Mol Cell Biol.

[12] Courtheoux T, Gay G, Gachet Y, Tournier S (2009). Ase1/Prc1-dependent spindle elongation corrects merotely during anaphase in fission yeast.. J Cell Biol.

[13] Farr KA, Hoyt MA (1998). Bub1p kinase activates the Saccharomyces cerevisiae spindle assembly checkpoint.. Mol Cell Biol.

[14] Waters JC, Chen RH, Murray AW, Salmon ED (1998). Localization of Mad2 to kinetochores depends on microtubule attachment, not tension.. J Cell Biol.

[15] Beinhauer JD, Hagan IM, Hegemann JH, Fleig U (1997). Mal3, the fission yeast homologue of the human APC-interacting protein EB-1 is required for microtubule integrity and the maintenance of cell form.. J Cell Biol.

[16] Tanaka K, Hirota T (2009). Chromosome segregation machinery and cancer.. Cancer Sci.

[17] Asakawa K, Toda T (2006). Cooperation of EB1-Mal3 and the Bub1 spindle checkpoint.. Cell Cycle.

[18] Pinsky BA, Biggins S (2005). The spindle checkpoint: tension versus attachment.. Trends Cell Biol.

[19] Beach DL, Thibodeaux J, Maddox P, Yeh E, Bloom K (2000). The role of the proteins Kar9 and Myo2 in orienting the mitotic spindle of budding yeast.. Curr Biol.

[20] Green RA, Wollman R, Kaplan KB (2005). APC and EB1 function together in mitosis to regulate spindle dynamics and chromosome alignment.. Mol Biol Cell.

[21] Dujardin D, Wacker UI, Moreau A, Schroer TA, Rickard JE (1998). Evidence for a role of CLIP-170 in the establishment of metaphase chromosome alignment.. J Cell Biol.

[22] Nabeshima K, Nakagawa T, Straight AF, Murray A, Chikashige Y (1998). Dynamics of centromeres during metaphase-anaphase transition in fission yeast: Dis1 is implicated in force balance in metaphase bipolar spindle.. Mol Biol Cell.

[23] Courtheoux T, Gay G, Reyes C, Goldstone S, Gachet Y (2007). Dynein participates in chromosome segregation in fission yeast.. Biol Cell.

[24] Busch KE, Brunner D (2004). The microtubule plus end-tracking proteins mal3p and tip1p cooperate for cell-end targeting of interphase microtubules.. Curr Biol.

[25] Javerzat JP, Cranston G, Allshire RC (1996). Fission yeast genes which disrupt mitotic chromosome segregation when overexpressed.. Nucleic Acids Res.

[26] Gachet Y, Tournier S, Millar JB, Hyams JS (2004). Mechanism controlling perpendicular alignment of the spindle to the axis of cell division in fission yeast.. EMBO J.

[27] Mallavarapu A, Sawin K, Mitchison T (1999). A switch in microtubule dynamics at the onset of anaphase B in the mitotic spindle of Schizosaccharomyces pombe.. Curr Biol.

[28] Tolic-Norrelykke IM, Sacconi L, Thon G, Pavone FS (2004). Positioning and elongation of the fission yeast spindle by microtubule-based pushing.. Curr Biol.

[29] Gardner MK, Haase J, Mythreye K, Molk JN, Anderson M (2008). The microtubule-based motor Kar3 and plus end-binding protein Bim1 provide structural support for the anaphase spindle.. J Cell Biol.

[30] Pidoux AL, Uzawa S, Perry PE, Cande WZ, Allshire RC (2000). Live analysis of lagging chromosomes during anaphase and their effect on spindle elongation rate in fission yeast.. J Cell Sci.

[31] Draviam VM, Shapiro I, Aldridge B, Sorger PK (2006). Misorientation and reduced stretching of aligned sister kinetochores promote chromosome missegregation in EB1- or APC-depleted cells.. EMBO J.

[32] Grancell A, Sorger PK (1998). Chromosome movement: kinetochores motor along.. Curr Biol.

[33] Molk JN, Salmon ED, Bloom K (2006). Nuclear congression is driven by cytoplasmic microtubule plus end interactions in S. cerevisiae.. J Cell Biol.

[34] Narumiya S, Yasuda S (2006). Rho GTPases in animal cell mitosis.. Curr Opin Cell Biol.

[35] Tournier S, Gachet Y, Buck V, Hyams JS, Millar JB (2004). Disruption of astral microtubule contact with the cell cortex activates a Bub1, Bub3, and Mad3-dependent checkpoint in fission yeast.. Mol Biol Cell.

[36] Wuhr M, Mitchison TJ, Field CM (2008). Mitosis: new roles for myosin-X and actin at the spindle.. Curr Biol.

[37] Lenart P, Bacher CP, Daigle N, Hand AR, Eils R (2005). A contractile nuclear actin network drives chromosome congression in oocytes.. Nature.

[38] Rogers SL, Rogers GC, Sharp DJ, Vale RD (2002). Drosophila EB1 is important for proper assembly, dynamics, and positioning of the mitotic spindle.. J Cell Biol.

[39] Muhua L, Adames NR, Murphy MD, Shields CR, Cooper JA (1998). A cytokinesis checkpoint requiring the yeast homologue of an APC-binding protein.. Nature.

[40] Tanenbaum ME, Galjart N, van Vugt MA, Medema RH (2006). CLIP-170 facilitates the formation of kinetochore-microtubule attachments.. EMBO J.

[41] Dzhindzhev NS, Rogers SL, Vale RD, Ohkura H (2005). Distinct mechanisms govern the localisation of Drosophila CLIP-190 to unattached kinetochores and microtubule plus-ends.. J Cell Sci.

[42] He X, Rines DR, Espelin CW, Sorger PK (2001). Molecular analysis of kinetochore-microtubule attachment in budding yeast.. Cell.

[43] Wolyniak MJ, Blake-Hodek K, Kosco K, Hwang E, You L (2006). The regulation of microtubule dynamics in Saccharomyces cerevisiae by three interacting plus-end tracking proteins.. Mol Biol Cell.

[44] Miller RK, Heller KK, Frisen L, Wallack DL, Loayza D (1998). The kinesin-related proteins, Kip2p and Kip3p, function differently in nuclear migration in yeast.. Mol Biol Cell.

[45] Sheeman B, Carvalho P, Sagot I, Geiser J, Kho D (2003). Determinants of S. cerevisiae dynein localization and activation: implications for the mechanism of spindle positioning.. Curr Biol.

[46] Grishchuk EL, Spiridonov IS, McIntosh JR (2007). Mitotic chromosome biorientation in fission yeast is enhanced by dynein and a minus-end-directed, kinesin-like protein.. Mol Biol Cell.

[47] Gachet Y, Reyes C, Courtheoux T, Goldstone S, Gay G (2008). Sister kinetochore recapture in fission yeast occurs by two distinct mechanisms, both requiring dam1 and klp2.. Mol Biol Cell.

[48] West RR, Malmstrom T, Troxell CL, McIntosh JR (2001). Two related kinesins, klp5+ and klp6+, foster microtubule disassembly and are required for meiosis in fission yeast.. Mol Biol Cell.

[49] Grishchuk EL, McIntosh JR (2006). Microtubule depolymerization can drive poleward chromosome motion in fission yeast.. EMBO J.

